# Supplementary material for: DMRforPairs: identifying Differentially Methylated Regions between unique samples using array based methylation profiles
Source: BMC Bioinformatics. 2014 May 15;15:141. doi: 10.1186/1471-2105-15-141 (PMC4046028; doi:10.1186/1471-2105-15-141)
Supplement: Additional file 2 — DMRforPairs output for the comparison of A431-MCF7 and NA17018-NA17105. Please start from the HTML files in each folder. Available via the BMC Bioinformatics website. [file 1471-2105-15-141-S2.zip › 1394847754114233_MOESM2_ESM/A431_MCF7/figures/16929.pdf]

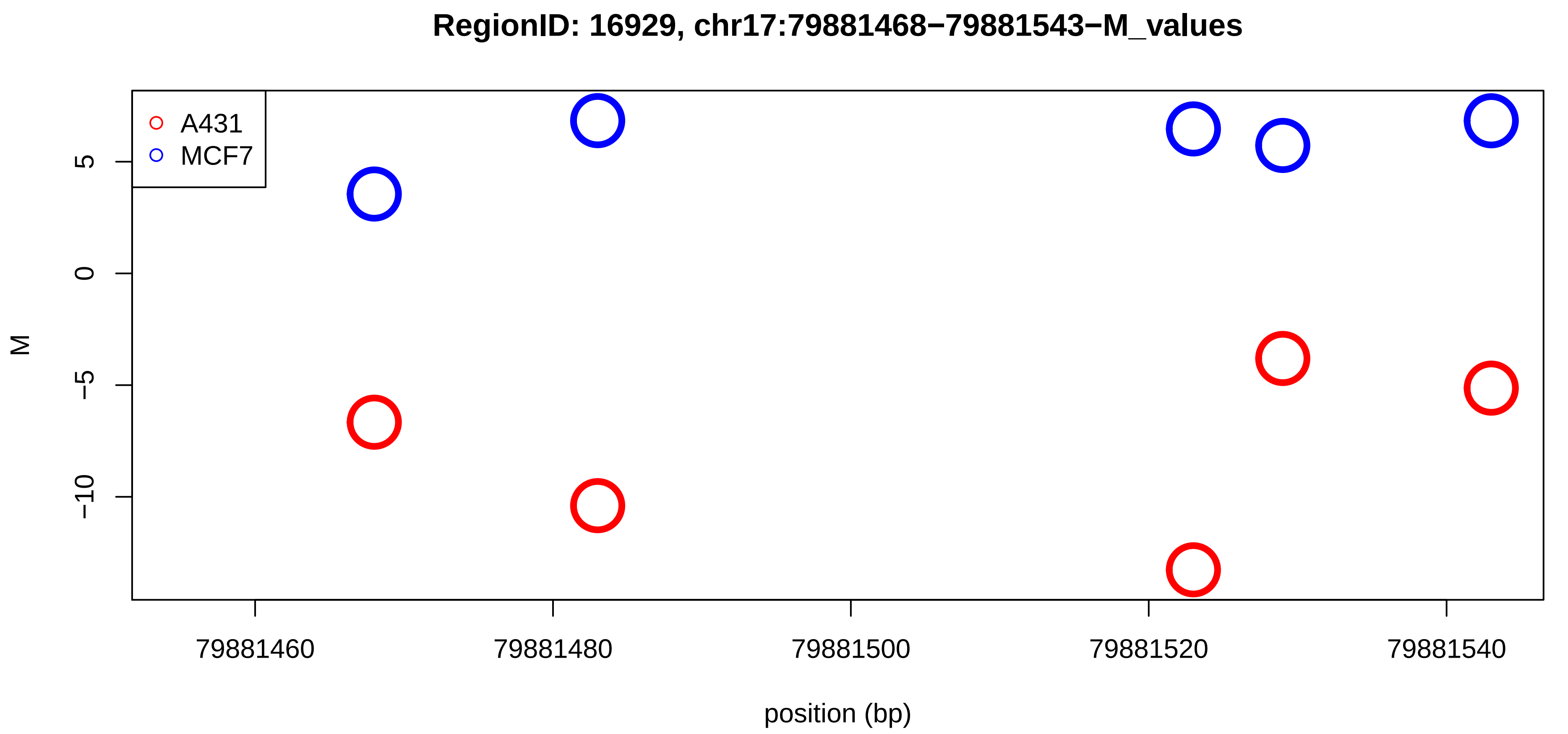
RegionID: 16929, chr17:79881468−79881543−M_values
A431
MCF7
M
5
0
−5
−10
79881460
79881480
79881500
79881520
79881540
position (bp)

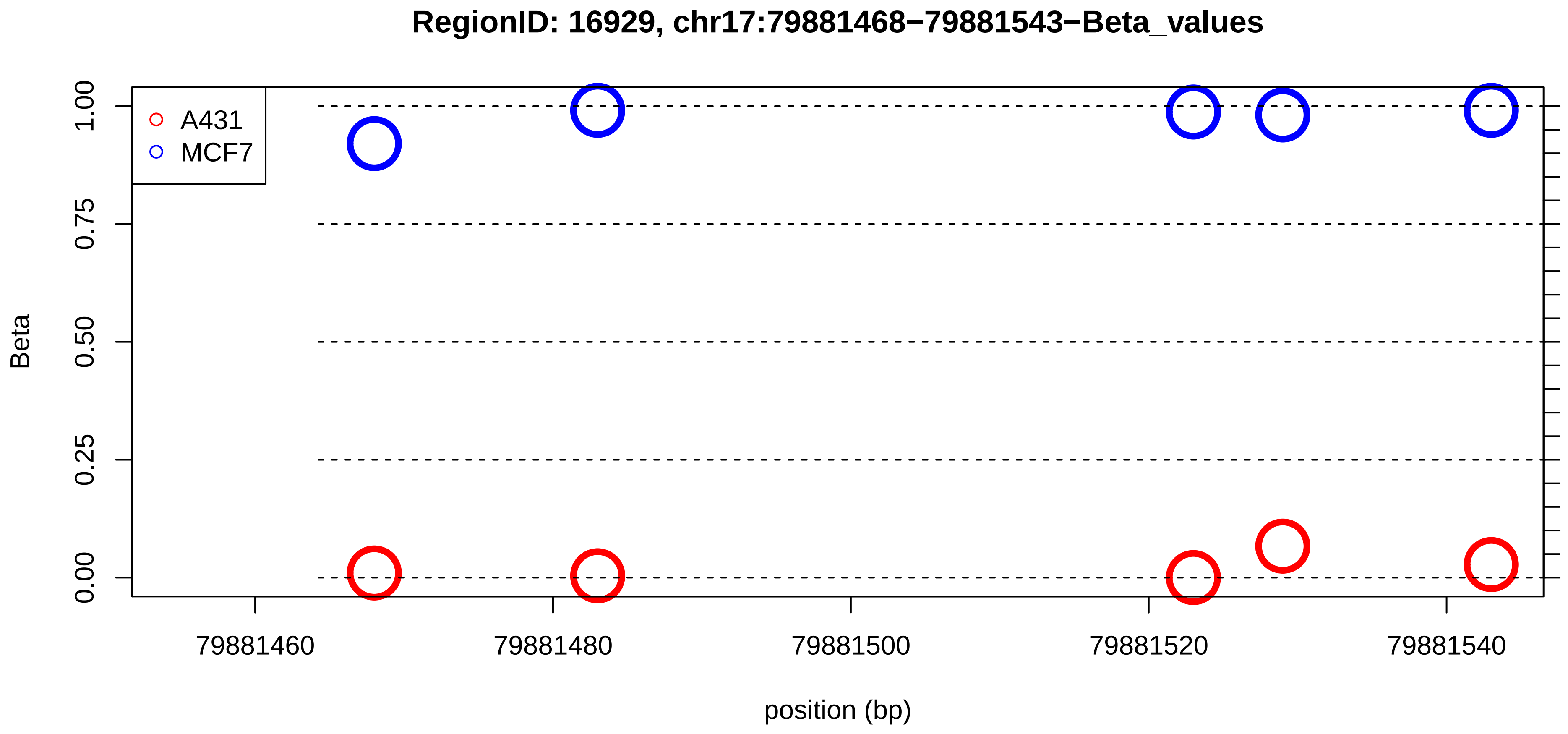
RegionID: 16929, chr17:79881468−79881543−Beta_values
A431
MCF7
Beta
1.00
0.75
0.50
0.25
0.00
79881460
79881480
79881500
79881520
79881540
position (bp)

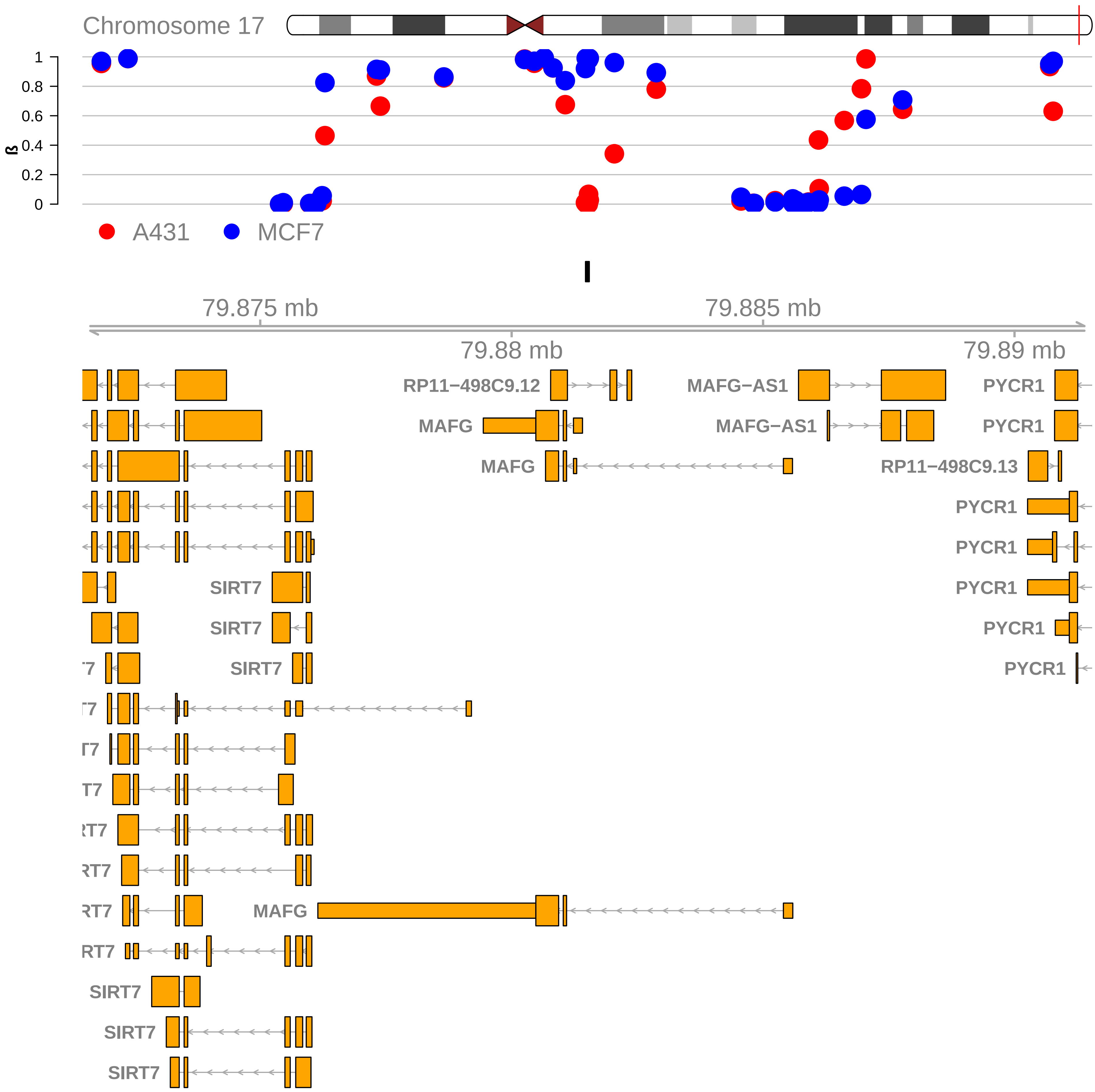

Chromosome 17
1
0.8
0.6
ß 0.4
0.2
0
A431
MCF7
79.875 mb
79.885 mb
79.88 mb
79.89 mb
RP11−498C9.12
MAFG−AS1
PYCR1
MAFG
MAFG−AS1
PYCR1
MAFG
RP11−498C9.13
PYCR1
PYCR1
SIRT7
PYCR1
SIRT7
PYCR1
SIRT7
PYCR1
MAFG
SIRT7
SIRT7
SIRT7
